# COVID-19 Vaccine Intentions and Perceptions Among Public School Staff of the Greater Vancouver Metropolitan Area, British Columbia, Canada

**DOI:** 10.3389/fpubh.2022.832444

**Published:** 2022-04-27

**Authors:** Allison W. Watts, Sarah M. Hutchison, Julie A. Bettinger, Anne Gadermann, Eva Oberle, Tim F. Oberlander, David M. Goldfarb, Pascal M. Lavoie, Louise C. Mâsse

**Affiliations:** ^1^Department of Pediatrics, Faculty of Medicine, University of British Columbia, Vancouver, BC, Canada; ^2^British Columbia Children's Hospital Research Institute, University of British Columbia, Vancouver, BC, Canada; ^3^Vaccine Evaluation Center, British Columbia Children's Hospital Research Institute, BC Children's Hospital, Vancouver, BC, Canada; ^4^Human Early Learning Partnership, School of Population and Public Health, University of British Columbia, Vancouver, BC, Canada; ^5^Centre for Health Evaluation and Outcome Sciences, Providence Health Care Research Institute, St. Paul's Hospital, Vancouver, BC, Canada; ^6^British Columbia Children's and Women's Health Centre, Vancouver, BC, Canada; ^7^School of Population and Public Health, University of British Columbia, Vancouver, BC, Canada

**Keywords:** COVID-19, vaccine hesitancy, teachers, schools, vaccine intention

## Abstract

**Background:**

The purpose of this study was to explore factors associated with COVID-19 vaccine intentions among school staff as high vaccine uptake is essential to ensure schools return to normal activities.

**Methods:**

Staff (e.g., teachers, administrators, student support workers) from three urban school districts in the Greater Vancouver Area of British Columbia, Canada completed a survey between February and June 2021 (*n* = 2,393) on COVID-19 vaccine intentions and perceptions (i.e., acceptance of routine vaccines, benefits and risks of vaccination, susceptibility to, and severity of COVID-19, recommendation by authority figures, information mistrust and conspiracy beliefs) as part of a COVID-19 seroprevalence study. Confirmatory factor analysis followed by multiple logistic regression models adjusting for relevant covariates were used to identify vaccine perceptions uniquely associated with (a) intention to get the COVID-19 vaccine (*intention*), and (b) intention to get vaccinated right away (*urgency*).

**Results:**

In total, 95.4% of participants of the seroprevalence study completed the vaccine questionnaire, corresponding to 17.7% of the target population. Vaccine *intention* was associated with staff who valued expert recommendations (AOR = 10.5, 95% CI = 7.39–14.90) accepted routine vaccines (AOR = 1.94, 95% CI = 1.26–2.98) and perceived higher benefits (AOR = 1.29, 95% CI = 1.01–1.65) and lower safety risks of vaccination (AOR = 0.40, 95% CI = 0.29–0.54). Comparable associations were found with vaccine *urgency*. Perceived susceptibility to the COVID-19 virus was uniquely associated with vaccine *urgency* (AOR = 1.30, 95% CI = 1.05–1.61). A significant interaction effect (*p* = 0.01) revealed that staff who expressed mistrust in COVID-19 information intended to get vaccinated only if they also perceived high benefits of vaccination.

**Conclusions:**

Education about the risks and benefits of COVID-19 vaccines from a trusted source had the strongest relationship with vaccine intentions among this occupational group. Notably, those who expressed mistrust in information still intended to get vaccinated if they also perceived strong benefits of the vaccine.

## Introduction

High vaccine uptake is necessary to reduce community transmission of SARS-CoV-2, yet vaccine acceptance stagnates in Western nations despite accumulating evidence supporting the safety and effectiveness of COVID-19 vaccines. Teachers play a critical role in educating youth and supporting the economy and as such they are a priority group to avoid further closures or disruptions in the school system which has broad social and economic implications (e.g., disrupted learning, gaps in childcare, strain on all essential workers) (1). High vaccine uptake is particularly important among this occupational group, to allow in-person activities to safely take place in schools. Furthermore, there has been considerable public discussion and media attention around safety within schools about COVID-19. Even though school staff are perceived to be at high risk of infection for COVID-19, there is still a significant proportion who are hesitant to be vaccinated, which is estimated to be over 12% based on US data (2) and around 10% based on Canadian data (3). Therefore, return to “normal” schooling plans must address the individual, social and ideological factors that contribute to vaccine intentions among school staff.

Studies that have examined factors associated with vaccine intentions have typically focused on understanding those who get the vaccine vs. those who do not get it instead of viewing vaccine intentions on a continuum (4). Such perspectives highlight that the factors associated with COVID-19 vaccine intention may differ among those who opt to get the vaccine right away vs. those who express wanting to get the vaccine later (4). For example, while negative attitudes and concerns about COVID-19 vaccines were found to be higher among those who use social media as their main source of information (5), it remains unclear whether these attitudes and concerns predict the intention and urgency of vaccine uptake. Taking a nuanced approach to understand vaccine intentions among school staff could best inform public health campaigns aimed at increasing vaccine acceptance among school staff.

Given the limited literature on COVID-19 vaccine perceptions and intentions among school staff, this study analyzed quantitative data from three large urban school districts in the Vancouver Metropolitan region of Canada, to explore the perceptions associated with COVID-19 vaccine intentions before the broad introduction of COVID-19 vaccines in the Canadian population. To better understand the nuances of vaccine intentions, this study examined the factors that were uniquely associated with (a) intention to get the COVID-19 vaccine (*Vaccine Intention*) and (b) intention to get the COVID-19 vaccine right away (*Vaccine Urgency*).

**Figure 1:**
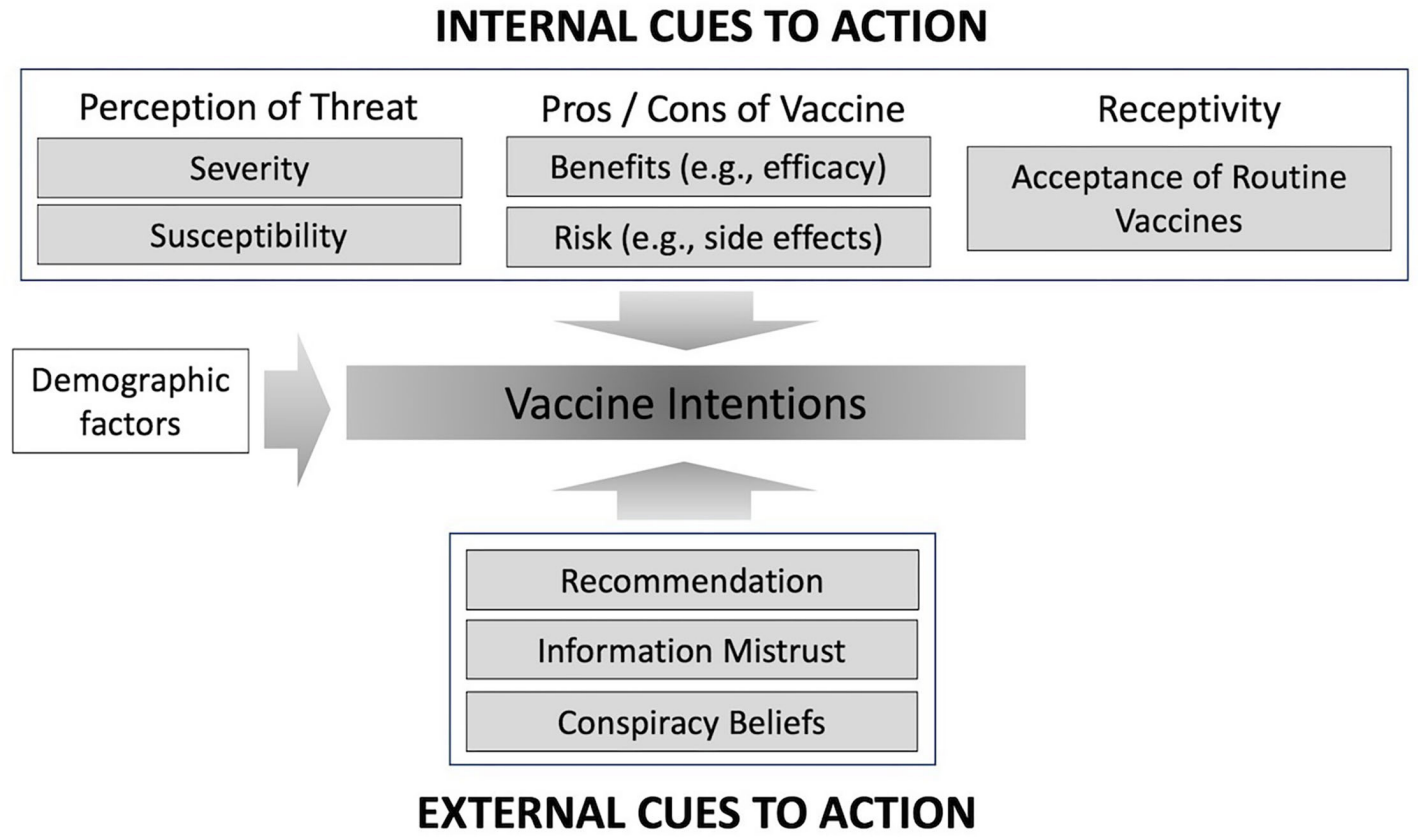
Factors hypothesized to be associated with COVID-19 vaccine intentions.

The Health Belief Model (HBM) offered a well-tested framework for understanding vaccine intentions (6) and was predominantly used to select the factors investigated in this study (Figure 1). Applying the HBM to COVID-19, individuals are hypothesized to be more likely to be vaccinated if they believe they are more susceptible to COVID-19, that COVID-19 is a serious condition, and that vaccination would reduce their susceptibility to infection. In addition, the model posits that vaccine intention is more likely for individuals who are confident in their ability to obtain a vaccine, or for individuals who believe COVID-19 vaccines do not incur excessive personal costs, and the benefits of vaccination outweigh these costs (7, 8). In this regard, British Columbia (BC) was the jurisdiction in Canada that maintained in-person school activities for the longest period of time throughout the 2020/2021 school year, which may have worsened the perception of a COVID-19 infection risk among the school staff. In addition, vaccine misconceptions and mistrust of information were added to the HBM as these two factors have been associated with vaccine refusal or acceptance (5, 9–11) (Figure 1).

## Methods

### Participants and Procedures

Current, active staff members of three school districts in the Greater Vancouver Metropolitan Area were surveyed about vaccine intention as part of a seroprevalence study (12). District staff were recruited in the main seroprevalence study through an advertisement posted to the district websites and emailed directly to staff by school principals, inviting them to self-register online via the study website. Staff were ineligible if they indicated that they were temporary staff, on-leave, or who identified as a teacher-on-call without any classroom time. Staff who were invited to participate could have been classroom teachers or support workers, school administrators, other staff in the school including maintenance staff, as well as individuals working in the school district administrative office. To complete enrollment in the study, participants were assessed for eligibility with an online screening tool, provided their contact information including a school district email address to verify eligibility, watched a video about the study were emailed a copy of the consent form, and provided their consent. After consenting, participants were emailed a link to an online survey using a secure REDCap platform (13) that included a vaccine perceptions questionnaire. Participants received a $20 electronic gift card to a bookstore as a token of appreciation for their participation. The study was approved by the University of British Columbia Children's and Women's Research Ethics Board (H20-03593).

### Context

Participants enrolled in the study between February 3 and June 18, 2021. The participating BC school districts remained open during the 2020/2021 school year where elementary schools maintained 100% in-person learning and high schools had a combination of in-person and online learning for all students. Vaccination of high-risk elderly in long-term care facilities started in mid-December, 2020. However, given the limited vaccine supply available in Canada, access was restricted to those at greatest risk for severe outcomes (e.g., the elderly) and front-line health care workers. District staff were not prioritized for vaccination until April 15^th^, 2021.

### Measures

The vaccine perception questionnaire was developed to capture vaccine intentions and predictors of vaccine intentions based on the domains of the HBM (6), additional constructs hypothesized to be related to vaccine intentions (14–18) (see Figure 1), and personal demographic and occupational characteristics. The full questionnaire is presented in Appendix A.

Vaccine Perception survey items are presented in **Table 2** also details how vaccine perception survey items were combined into scales and presents the psychometric properties of each scale, validated through confirmatory factor analyses. All scales had high internal consistency (Cronbach alphas ranging from 0.72 to 0.86). Scale scores were calculated as the mean of the items within each scale and were standardized on a scale from 1 to 5, whereby 5 indicates stronger agreement with the construct. To create scale scores, survey item response options were “reversed” to match the direction of the other items within the same scale.

Vaccine Intentions were measured with two items from the WHO Behavioral insights on COVID-19 survey tool (13): Vaccine Intention was measured using an item, “*If a vaccine becomes available and is recommended for me, I would get it*” (*Yes/No/Unsure*); while Vaccine Urgency captured vaccine intention with urgency and was measured using the item, “*If I were to be vaccinated, I would get it as soon as it would be available to me*” (*Yes, right away/Yes, but I would wait a little bit/No, I don't plan on getting it/Unsure*).

### Statistical Analyses

Descriptive data were examined and are presented as frequency and proportion, mean and standard deviation, or median and interquartile range, as appropriate (Tables 1, 2). Prior to conducting the main analyses, the psychometric properties of the measures were assessed by examining internal consistencies and by conducting Confirmatory Factor Analysis (CFA) (Table 2). Internal consistency of the measures was determined by computing Cronbach's Alpha. The CFA assessed if the factor structures of these measures were supported in the analytical sample. Model fit was assessed using commonly accepted fit indices for CFA (19, 20): Chi-square goodness of fit test (*p*-value ≥ 0.15), Comparative Fit Index (CFI > 0.95), Root Mean Square Error of Approximation (RMSEA < 0.08), and the Standardized Root Mean Square Residual (SRMR < 0.08).

**Table 1:** Characteristics of participating school district staff (*n* = 2,393)^a^.

**Characteristic**	***n* (%)**	**Mean (SD) [IQR]**
**Sex**, female	1,953 (81.9%)	
**Age**, yrs		45.4 (10.4) [38.0–53.0]
**Ethnicity**		
White/European	1,627 (68.7%)	
East Asian	396 (16.7%)	
South/West Asian	169 (7.1%)	
Mixed ethnicity/Other	135 (5.7%)	
Indigenous	42 (1.8%)	
**Occupation**		
Teacher	1,434 (60.0%)	
Student support/Youth & family workers	373 (15.6%)	
Administration (principal, office staff)	250 (10.5%)	
Other (e.g., Board office, maintenance, other)	335 (14.0%)	
**School level**		
Elementary	1,436 (60.0%)	
Secondary	760 (31.8%)	
Work at multiple, mixed level schools	82 (3.4%)	
School board office only	114 (4.8%)	
**Education**		
< University/College degree	145 (6.1%)	
Community college diploma	273 (11.4%)	
University bachelor's degree	1,102 (46.2%)	
Graduate degree	868 (36.4%)	
**Exposure to students**, average hours/wk		18.4 (11.8) [5.0–25.0]
**Living with 1+** **essential worker** (not including self)	951 (40.1%)	
**Has a chronic medical condition** ^b^	596 (24.9%)	
**Received flu shot in 2020**, % Yes	1,891 (79.1%)	
**Intention to be vaccinated**		
Yes	2,214 (92.5%)	
No	11 (0.5%)	
Maybe	168 (7.0%)	
**Urgency to be vaccinated**		
Yes, right away	1,973 (82.4%)	
Yes, but I will wait	317 (13.3%)	
No	9 (0.4%)	
Unsure	96 (4.0%)	

a *Small amount of missing data varies by variable (range n = 2,369–2,393)*.

b *Chronic medical condition, those who selected one or more of the following conditions: hypertension, diabetes, asthma, chronic heart/ lung/ kidney disease, liver disease, cancer, chronic blood disorder, immune suppressed, chronic neurological disorders*.

*SD, Standard Deviation; IQR, Interquartile Range*.

**Table 2:** Vaccine perceptions scale scores and psychometric properties assessed by confirmatory factor analysis (CFA).

**Scale**	***n***	**Mean [Median (IQR)]^a^**	**Items**	**Standardized factor loading**	**Chronbach's alpha**
**Model 1:** 1-factor CFA regrouping 6 items to measure receptivity to routine vaccines: *χ^2^(df = 9) = 133, p < 0.01; RMSEA = 0.08 [0.07–0.09], p < 0.01; CFI = 0.97; and SRMR = 0.03*					
Acceptance of routine vaccines (16)	2,393	4.5 [4.7 (4.2–5.0)]	I am completely confident that routine vaccines are safe	0.70	α = 0.78
			Routine vaccination is unnecessary because vaccine-preventable diseases are not common anymore	0.61	
			When everyone else is vaccinated, I don't have to get vaccinated too	0.67	
			People should be vaccinated to prevent the spread of disease in the community	0.65	
			Everyday stress (such as competing priorities or many demands on my time) prevents me from getting vaccinated	0.43	
			Vaccines are effective	0.69	
**Model 2:** 5-factor CFA regrouping the constructs of the Health Belief Model^b^ : *χ^2^(df = 94) = 532, p < 0.01; RMSEA = 0.045 [0.04–0.05], p = 0.99; CFI = 0.97; and SRMR = 0.03*					
Value expert recommendations (15)	2,393	4.6 [5.0 (4.3–5.0)]	I would get the COVID-19 vaccine if my healthcare provider recommends it	0.93	α = 0.74
			I would get the COVID-19 vaccine if public health experts recommend it	0.84	
			I would get the COVID-19 vaccine if the government recommends it	0.56	
Perceived benefits of the vaccine	2,391	4.1 [4.3 (3.0–5.0)]	Receiving the COVID-19 vaccine would: Protect me from getting COVID-19	0.88	α = 0.79
			Protect my family from getting COVID-19	0.87	
			End the pandemic and make us return to normal life	0.54	
Perceived susceptibility to the virus	2,393	3.7 [3.8 (3.3–4.3)]	I am at risk of getting COVID-19	0.73	α = 0.72
			Someone in my family is at risk of getting COVID-19	0.77	
			I am at risk of severe complications from COVID-19	0.47	
			Someone in my family is at risk of getting really sick from COVID-19	0.58	
Perceived severity of the virus	2,393	4.6 [5.0 (4.3–5.0)]	COVID-19 is a serious disease	0.90	α = 0.85
			People can die if they get COVID-19	0.88	
			People who have mild symptoms for COVID-19, can still have long term health effects	0.68	
Perceived risk of the vaccine	2,392	2.8 [2.7 (2.0–3.7)]	The following may prevent me from getting the vaccine:		
			It may have serious short term side effects	0.81	α = 0.87
			It may have long term effects that we are unaware of	0.92	
			We do not know whether it will protect us for a long time	0.77	
**Model 3:** 2-factor CFA regrouping the constructs about trusting external information^c^: *χ^2^(df = 24) = 264, p < 0.01; RMSEA = 0.07 [0.06–0.07], p < 01; CFI = 0.98; and SRMR = 0.04*					
Mistrust of COVID-19 information (14, 17)	2,360	2.1 [2.0 (1.3–2.7)]	Much of the information we receive about COVID-19 is wrong	0.70	α = 0.86
			I think health officials often hide the truth about COVID-19	0.89	
			Official government accounts of COVID-19 cannot be trusted	0.90	
Belief in conspiracies (14, 17)	2,377	2.4 [2.4 (1.8–3.0)]	I believe the coronavirus was created in a laboratory according to plans unknown to the public	0.54	α = 0.82
			I believe there are groups interested in spreading panic about COVID-19 to achieve their own goals	0.43	
			Many very important things happen in the world, which the public is never informed about	0.65	
			Politicians usually do not tell us the true motives in their decisions	0.73	
			Events which superficially seem to lack a connection are often the result of secret activities	0.75	
			There are secret organizations that greatly influence political decisions	0.74	

a *Scale scores are standardized from 1 to 5, representing low to high scores for each construct*.

b *Correlations between the Health Belief Model latent variables were as follows: r = 0.08 (benefits & risk), 0.16 (benefits & severity),−0.24 (benefits & barriers), 0.37 (benefits & recommendation), 0.47 (risks & severity), −0.07 (risk & barriers), 0.17 (risk & recommendation), −0.18 (severity & barriers), 0.27 (severity & recommendation), −0.37 (barriers & recommendation)*.

c *Model 3 included a correlated error term among items within the Belief in Conspiracy measures include between items 3 & 4 (r = 0.43) and between items 5 & 6 (r = 0.44)*.

*IQR, Interquartile Range*.

Separate logistic regression models were used to examine associations between vaccine-related perceptions and each outcome measure of vaccine intention: (a) intention to get the COVID-19 vaccine (*vaccine intention*) and (b) intention to get the vaccine urgently/right away (*vaccine urgency*). For these analyses, *vaccine intention* was dichotomized as 1 = Yes, I will get vaccinated vs. 0 = No/Unsure and *vaccine urgency* was dichotomized as 1 = Yes, I will get the vaccine right away vs. 0 = Yes, but I will wait/No/Unsure.

Personal factors including *age, sex, ethnicity, education level* and *number of hours in contact with students* were hypothesized to be related to vaccine intentions and were included in each model as covariates. For the analysis, *ethnicity* was categorized as 0 = White (reference)/1 = East Asian/2 = South or West Asian/3 = other or did not answer, and *education* was dichotomized as 0 = university degree (reference)/1 = less than a university degree. Associations between each independent or covariate variable and outcomes were first examined in unadjusted, bivariable models. Second, all independent and covariate variables were included simultaneously in a mutually adjusted model. Finally, a descriptive examination and comparison of the two mutually adjusted models was done by identifying similarities or differences in the presence, strength and direction of statistically significant associations. Multicollinearity between variables was examined using Variance Inflation Factors and all were found to be <2. Results are presented as Odds Ratios (OR) for bivariable models or Adjusted Odds Ratios (AOR) for mutually adjusted models and 95% Confidence Intervals (95% CI). All statistical tests were two-sided, with *p* < 0.05 considered statistically significant. All analyses were conducted using Stata Statistical Software, version 16 (21).

As a sensitivity analysis, missing data (<5% variables) were imputed using multiple imputation with chained equations (MICE) and 10 imputations. Outcome variables were included in the imputation but observations missing on the outcome variable were dropped prior to imputed regression analyses. No changes to the significance or magnitude of the results were found (data not shown). Those with missing data on vaccine intentions and/or >15% missing data across vaccine perception questions were removed from the analytic sample, and remaining analyses were conducted using complete case analysis.

Targeted *post-hoc* interaction effects between independent variables and vaccine intention were examined to investigate associations that changed direction after including all independent variables into the model. Interactions between the variables of interest and the other model variables were examined in mutually adjusted models and those with a *p*-value <0.10 were examined in mutually adjusted stratified regression models.

## Results

### Sample Characteristics

In total, 3,315 staff members were assessed for eligibility through the online screening tool and 2,538 consented to the parent seroprevalence study. Of these (*n* = 2,538), the vaccine perceptions questionnaire was completed by 2,421 staff (95.4% response rate); 28 were excluded because they did not complete items on vaccine intentions and/or had >15% missing data across the survey questions. We estimated that a total of 13,517 eligible staff were invited to participate to the parent seroprevalence study; therefore, 2,393 participating staff represent 17.7% of eligible staff across the three school districts. Human resources data obtained from the 2 out of the 3 participating school districts show that the age and sex of respondents to the current vaccine survey were representative of the target population (target population had a mean age of 46.4 yrs and 73.6% were female). The sample was also previously reported to be representative of the target population based on geographical residence and COVID-19 positivity (12).

The characteristics of study participants are presented in Table 1. A majority were females of white/European or Asian descent, with a mean age of 45 years (range 19 to 79 years) and 82.6% had a university bachelor's degree. The distribution of staff from elementary/secondary schools was proportional to the district population where 82% of schools are elementary schools. About 75.6% of respondents worked directly in a classroom setting as a teacher, student support worker or youth & family worker, with an overall average direct contact time with students of 18.4 h per week. Respondents also included school administrative staff (10.5%) or those who work at the district office, as maintenance workers or others (14%).

### COVID-19 Vaccine Intentions and Perceptions

A majority of staff reported they intend to be vaccinated (92.5%) and that they will get vaccinated right away (82.4%) (Table 1). Table 2 reports the vaccine-related perceptions of the school staff and the psychometric analyses of these measures. High median scores were observed for *receptivity to routine vaccines* (M = 4.7, IQR = 4.2–5.0), perceived *value of expert recommendations* (M = 5.0, IQR = 4.3–5.0), *perceived severity of the virus* (M = 5.0, IQR = 4.3–5.0), and *perceived benefits of the vaccine* (M = 4.3, IQR = 3.0–5.0). *Perceived susceptibility to the vaccine* was moderate among this group (M = 3.8, IQR = 3.3–4.3), while scores on the *perceived risk of the vaccine* (M = 2.7, IQR = 2.0–3.7), *mistrust in COVID-19 information* (M = 2.0, IQR = 1.3–2.7), and *belief in conspiracies* were low (M = 2.4, IQR = 1.8–3.0).

### Factors Associated With Intention to Get Vaccinated Against COVID-19

In unadjusted univariable models (Table 3), all of the HBM domains were associated with vaccine intention. Those significantly positively associated with *intention to be vaccinated* included higher *receptivity to routine vaccines, perceived value of expert recommendation* and greater *perceived severity of the virus, susceptibility to the virus*, and *perceived benefits of the vaccine*. Those significantly negatively associated with *intention to be vaccinated* included a greater *perceived risk of the vaccine, mistrust of COVID-19 information*, and *belief in conspiracies*.

**Table 3:** Univariable and multiple logistic regression models examining associations between personal factors, vaccine perceptions and COVID-19 vaccine intentions.

	**Intention (“Yes, I will get vaccinated” vs. “Unsure/No”)**		**Urgency (“Yes, as soon as possible” vs. “I will wait /Unsure/No”)**	
	**OR (95% CI)**	**AOR (95% CI)**	**OR (95% CI)**	**AOR (95% CI)**
**Personal factors**
Age	1.01 (0.99–1.02)	1.01 (0.99–1.04)	1.00 (0.99–1.02)	1.00 (0.99–1.02)
Sex (Female)	0.72 (0.47-−1.12)	0.99 (0.41–2.35)	**0.70 (0.50–0.99)**	0.78 (0.52–1.18)
Ethnicity
White	Reference	Reference	Reference	Reference
East Asian	**0.50 (0.35–0.73)**	0.73 (0.41–1.29)	**0.32 (0.24–0.44)**	**0.39 (0.27–0.57)**
South/West Asian	0.87 (0.34–1.38)	1.15 (0.48–2.79)	0.66 (0.40–1.09)	0.96 (0.56–1.65)
Other/Did not answer	0.60 (0.47–1.61)	0.72 (0.31–1.69)	**0.62 (0.40–0.97)**	0.88 (0.50–1.56)
Education (< University degree)	**0.48 (0.34–0.68)**	0.99 (0.57–1.75)	0.94 (0.54–1.64)	0.91 (0.63–1.32)
Hours exposed to students	0.99 (0.98–1.01)	1.00 (0.98–1.02)	**1.02 (1.01-1.03)**	**1.02 (1.01–1.03)**
**Vaccine perceptions**
Acceptance of routine vaccines	**7.54 (5.72–9.94)**	**1.94 (1.26–2.98)**	**7.33 (5.92–9.06)**	**2.69 (2.03−3.56)**
Value expert recommendations	**15.52 (11.56–20.85)**	**10.50 (7.39–14.9)**	**7.54 (6.24–9.10)**	**4.67 (3.71–5.87)**
Perceived severity of the virus	**2.30 (1.91–2.78)**	1.39 (0.92–2.11)	**2.22 (1.89–2.61)**	1.09 (0.84–1.42)
Perceived susceptibility to the virus	**1.62 (1.34–1.97)**	0.86 (0.59–1.25)	**1.64 (1.42–1.88)**	**1.30 (1.05–1.61)**
Perceived benefits of the vaccine	**2.61 (2.24–3.04)**	**1.29 (1.01–1.65)**	**2.11 (1.90–2.36)**	**1.36 (1.17–1.59)**
Perceived risk of vaccine	**0.27 (0.22–0.33)**	**0.40 (0.29−0.54)**	**0.34 (0.30–0.39)**	**0.54 (0.46–0.64)**
Mistrust of COVID-19 information	**0.37 (0.31–0.45)**	**1.69 (1.16–2.48)**	**0.41 (0.36–0.47)**	1.16 (0.93–1.46)
Belief in conspiracies	**0.36 (0.29–0.43)**	0.86 (0.59–1.25)	**0.37 (0.32–0.43)**	0.81 (0.65–1.03)

*N = 2,334 for multivariable model; OR, Odds Ratio; AOR, Adjusted Odds Ratio. Bolded values represent statistically significant associations at p < 0.05*.

In mutually adjusted models controlling for all covariates and vaccine perceptions (Table 3), *intention to be vaccinated* remained associated with *perceived value of expert recommendations* (AOR = 10.50, 95%CI = 7.39–14.9), higher *perceived benefits of the vaccine* (AOR = 1.29, 95%CI = 1.01–1.65) and lower *perceived risk of the vaccine* (AOR = 0.40, 95%CI = 0.29–0.54). Note that when the value of a recommendation by each type of source was explored separately in bivariate models, the value of a recommendation from each of *health providers, experts, and government* were all significantly associated with *intention to be vaccinated* and associations were highest for *experts* (OR = 10.0), then for *health providers* (OR = 7.6), followed by *government* (OR = 2.8). No personal factors were associated with vaccine intention in adjusted models.

**Figure 2:**
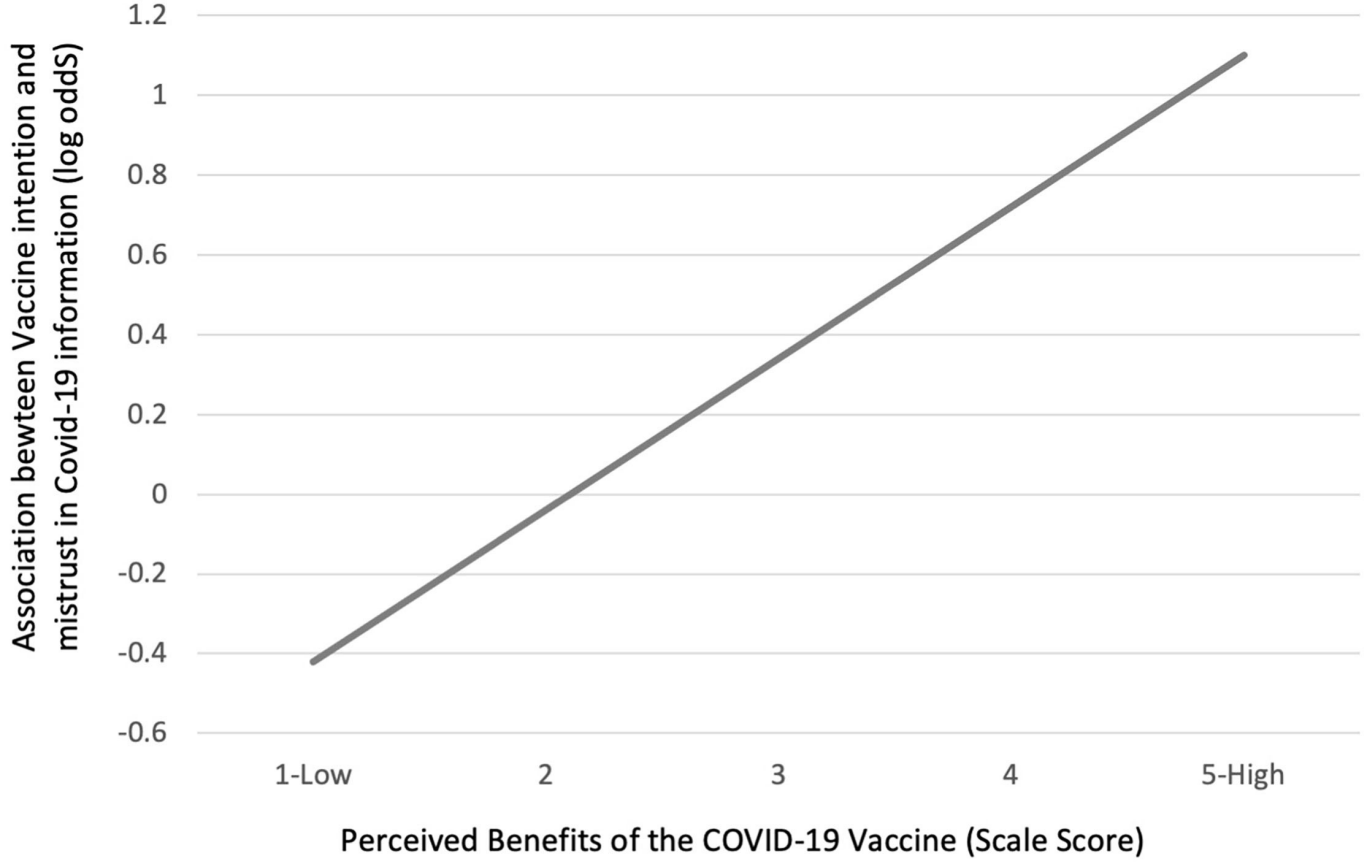
Interaction effects: adjusted associations between intentions to be vaccinated and level of mistrust in COVID-19 information (log odds) across levels of perceived benefits of the vaccine (1–5).

Unexpectedly, and in contrast to the bivariable associations, in mutually adjusted models, higher *mistrust of COVID-19 information* was positively associated with *intention to be vaccinated* (AOR = 1.69, 95%CI = 1.16–2.48). Interaction effects were examined to better understand this association. In a mutually adjusted model, a significant interaction was found between *perceived benefits of the vaccine* and *mistrust of COVID-19 information* (*p* = 0.01). Notably, *mistrust of COVID-19 information* was negatively associated with *intention to be vaccinated* when *perceived benefits of the vaccine* were low and positively associated when *perceived benefits of the vaccine* were high (Figure 2). In other words, individuals who perceived benefits of the vaccine to be high (to oneself, but also friends, family or society), still intended to get vaccinated despite a high level of mistrust in COVID-19 information.

### Factors Associated With Urgency to Get Vaccinated Against COVID-19

Similar to vaccine intention, all of the HBM domains were associated with vaccine urgency (i.e., intention to get the vaccine right away) in unadjusted univariable models. Those significantly positively associated with *urgency to be vaccinated* included higher *receptivity to routine vaccines, perceived value of expert recommendation* and greater *perceived severity of the virus, susceptibility to the virus*, and *perceived benefits of the vaccine*. Those significantly negatively associated with *urgency to be vaccinated* included a greater *perceived risk of the vaccine, mistrust of COVID-19 information*, and *belief in conspiracies* (Table 3).

In mutually adjusted models controlling for all covariates and vaccine perceptions (Table 3), *urgency to be vaccinated* was associated with greater *acceptance of routine vaccines* (AOR = 2.69, 95%CI = 2.03–3.56), greater *perceived value of expert recommendations* (AOR = 4.67, 95%CI = 3.71–5.87), greater *perceived susceptibility to the virus* (AOR = 1.30, 95%CI = 1-−1.61), greater *perceived benefits of the vaccine* (AOR = 1.36, 95%CI = 1.17–1.59) and lower *perceived risk of the vaccine* (AOR = 0.54, 95%CI = 0.46–0.64). In addition, reporting an *East Asian ethnicity* as compared to *White ethnicity* remained negatively associated with vaccine urgency (AOR = 0.39, 95%CI = 0.27–0.57) while greater number of *exposure hours to students* remained positively associated with *urgency to get the vaccine right away* in adjusted models (AOR = 1.02, 95%CI = 1.01–1.03).

## Discussion

This study gathered data on a continuum of vaccine intention and a range of vaccine-related perceptions among school staff who are an understudied, yet an essential occupational group for keeping schools open and for supporting the health and wellbeing of youth. A large majority of school staff surveyed from three urban school districts in British Columbia, Canada expressed that they intended to get vaccinated, and quickly. Recommendation by an expert, acceptance of routine vaccines low perceived risk of vaccination and strong belief in the benefits of vaccination were significantly associated with intention to get vaccinated. By taking a nuanced approach to examine vaccine intentions, this study uncovered that perceiving oneself at lower risk of contracting COVID-19 was uniquely associated with waiting to get vaccinated, while mistrust in COVID-19 information was uniquely associated with intention but not urgency to be vaccinated. Interestingly, intention to get vaccinated remained high even in those who expressed high mistrust in COVID-19 information if they also held a strong belief in the benefits of vaccination, suggesting that mistrust could be outweighed by strong public health messaging by experts. These novel findings based on data from a large urban center in Canada, may be useful to inform current and future vaccination campaigns among this occupational group.

Canadian data from the fall of 2020 found that vaccine safety, including the risk of the COVID-19 vaccines and possible side effects, were the most common reasons that Canadians were hesitant to be vaccinated (22). A previous study among BC school teachers found that earlier in the pandemic, vaccine knowledge and perceived severity of the virus predicted vaccine intention (3). These two previous studies took place before data on the safety and effectiveness of COVID-19 vaccines was available, whereas the present study collected data after the safety and effectiveness of COVID-19 vaccines were widely communicated by public health experts. In the present study, we found that valuing expert recommendations and confidence in the benefits and safety of vaccines remained dominant beliefs among those who intended to be vaccinated as compared to those who were unsure or did not intend to be vaccinated. The risk benefit ratio of the vaccine compared to infection may encourage vaccination when well communicated to school staff by a trusted figure. Findings suggested experts were most influential, followed by healthcare providers, then government. This is in line with research showing that reliable information from public health and healthcare providers can be important motivators of vaccine uptake among teachers and other groups (3, 23). On the other hand, research also shows that those who are hesitant of routine vaccines are less trusting of individuals involved in communicating vaccine recommendations including their health care provider and government sources or that it has no effect (17, 24). The impact of trusted information may differ depending on the population group, type (COVID-19 vs. routine vaccinates) and the target (adults vs. children) of the vaccine.

Some factors measuring perceived risk of infection were not related to overall intention to be vaccinated but were uniquely associated with how quickly school staff were willing to get vaccinated. The personal risk of contracting the virus was a dominant belief among those who wanted to get the vaccine right away. To increase uptake of the COVID-19 vaccine in the more hesitant group of individuals, public health messaging should explain the urgency of vaccine uptake among those who by virtue of their beliefs or role perceive themselves to be a low risk of contracting COVID-19. In addition, an East Asian ethnicity and the number of exposure hours to students were also associated with wanting to wait to be vaccinated. Exposure hours to students could be a proxy for perceived risk of infection. Those with less contact with students may perceive reduced exposure and lower perceived risk of infection thus influencing their decision to wait. Statistics Canada data show some differences in willingness to get vaccinated among population subgroups including higher intention among those identifying as South Asian and lower intention among those identifying as Black; no other differences were found (25).

When examined independently, belief in conspiracies and a mistrust in COVID-19 information were associated with lower intention and urgency to be vaccinated, however, these perceptions were low overall in this group who were supportive of vaccination and associations were attenuated by the addition of other attitudes and beliefs in the model. Based on analysis of interaction effects, a mistrust of COVID-19 information was positively associated with intention to get the COVID-19 vaccine only in the presence of high perceived benefits of the vaccine. This may suggest that the skeptical viewpoint held by some individuals may be outweighed by other positive benefits to themselves or the community, such as being able to return to normal activities. Research in the United States has reported that COVID-19 misinformation, conspiracy beliefs and mistrust may be countered by information from trusted and like-minded sources (26). Beliefs that vaccination will benefit self, family and the community may help to counter attitudes among those who are skeptical of COVID-19 information.

The proportion of school district staff who reported an intention to be vaccinated in the present study is in line with an October 2021 survey of 6,000 teachers across BC by the BC teachers federation. That study found that 94% of teachers had been vaccinated, a rate of vaccine uptake that is higher than the general population in BC (27). In early March 2021, Angus Reid reported that the highest rates of vaccine intention in BC were in Metro Vancouver (68%) as compared to 93% in the current study (the median survey completion date was March 12, 2021); although, intention was increasing over time in Canada and within BC (28). In the fall of 2020, 90% of BC school teachers reported they were likely or very likely to accept a COVID-19 vaccine (3), while a survey of educators in the US from February-March 2021, found that 85% had been vaccinated or intended to get vaccinated (29). The rate of seasonal flu vaccine uptake among the present sample of school staff was also much higher than the Canadian average of 42% reported during the 2019-2020 season (30), suggesting that educational staff in general, or participating school staff in this study, may engage in more health-seeking behaviors than the general population. Higher rates of vaccine intentions in our survey compared to the general population may be explained by differences in questionnaires, response rates or population groups. However, the high vaccine intention observed in this study is unlikely to be solely due to selection biases and is consistent with other findings among education workers (27).

This study had limitations to be considered when interpreting the findings. First, approximately 17% of eligible school district staff participated in the present study; however, the study was designed to capture a representative sample of district staff within a short and defined time period and had a high response rate (95.4%) among participants of the parent seroprevalence study. In addition, we found that the study sample was representative of the target population on age, sex, geographical residence and COVID-19 infection rates (12), although selection bias related to other criteria may influence vaccine intentions. Second, the current study focused on intentions to receive the first dose of the COVID-19 vaccines and it is not known how intentions may differ toward the second or subsequent doses or how emerging news of side effects (e.g., risk of blood clots) may impact hesitancy. Third, the epidemiology of COVID-19 cases changed over the course of the study and due to the cross-sectional nature of this study we were unable to analyze how vaccine perceptions were influenced by changes in COVID-19 case counts within schools or the community. The potential emergence of new variants may also have an influence on the perceived benefit of vaccination and perception of risks. Furthermore, the conceptual model examined in this study may have influenced the findings. Other, unmeasured constructs, including cultural pressures may also impact vaccine intentions. Finally, individuals in this sample were from an urban center, had a high level of education and were, in general, highly receptive to COVID-19 vaccination. Findings may have differed among those with lower education, from across geographical areas, from rural areas and with generally lower levels of vaccine receptiveness (22). For example, trusted information may not influence vaccine intentions in the same way in the US as compared to Canada.

In conclusion, domains of the HBM were related to both those who intend to get vaccinated and how quickly they intend to get vaccinated. The strongest staff intentions for getting vaccinated were related to the value placed on expert recommendations, followed by the perceived risks and benefits of vaccination and acceptance of routine vaccines. However, staff who chose to wait before getting vaccinated (but not staff with no intention to be vaccinated) perceived themselves as less susceptible to the virus. Despite the finding that school district staff had high intentions to get the COVID-19 vaccine overall, some were hesitant, and public health campaigns that educate individuals on their personal susceptibility to the virus may encourage those who are “on the fence” to get vaccinated sooner. In addition, communicating and emphasizing the risks and benefits of the current vaccines from trusted sources may be the best way to promote quick vaccine uptake among this group, and promoting strong beliefs in the benefits of vaccination may help to counter attitudes among those who are skeptical of COVID-19 information.

## Data Availability Statement

De-identified data used in these analyses will be made available by the authors upon request.

## Ethics Statement

The studies involving human participants were reviewed and approved by the University of British Columbia Children's and Women's Research Ethics Board (H20-03593). The patients/participants provided their written informed consent to participate in this study.

## Author Contributions

AW conducted the statistical analysis, interpreted the study results, and wrote the main manuscript text. SH, PL, and LM were involved in the study and instrument design, statistical analyses, interpreting results, and writing the manuscript. DG, JB, AG, EO, and TO were involved in the instrument design and interpretation of study results. All authors read and approved the study manuscript.

## Funding

The study was funded by the Public Health Agency of Canada through the COVID-19 Immunity Task Force. LM, PL, and TO receives a salary from the British Columbia Children's Hospital Research Institute (BCCHRI) through the Investigator Grant Award Program. The funder was not involved in the study design, analysis, interpretation or writing of manuscripts.

## Conflict of Interest

The authors declare that the research was conducted in the absence of any commercial or financial relationships that could be construed as a potential conflict of interest.

## Publisher's Note

All claims expressed in this article are solely those of the authors and do not necessarily represent those of their affiliated organizations, or those of the publisher, the editors and the reviewers. Any product that may be evaluated in this article, or claim that may be made by its manufacturer, is not guaranteed or endorsed by the publisher.
