# Supplementary material for: COVID-19 Vaccine Intentions and Perceptions Among Public School Staff of the Greater Vancouver Metropolitan Area, British Columbia, Canada
Source: Front Public Health. 2022 Apr 27;10:832444. doi: 10.3389/fpubh.2022.832444 (PMC9092339; doi:10.3389/fpubh.2022.832444)
Supplement: Supplementary file 1 [file Table_1.DOCX]

**Appendix A: Safer Schools Study Questionnaire**

| **Construct/Topic** | **Survey Question** | **Response format** |
| --- | --- | --- |
| **Height and weight** | 1. The next questions ask you to report how much you weigh. Would you like to report your weight in: | Pounds (lbs) / Kilograms (kg) |
|  | 1. What is your current weight in pounds? | 45-330 lbs |
|  | 1. What is your current weight in kilograms? | 20-150 kg |
|  | 1. The next questions ask you to report how tall you are. Would you like to report your height in: | Feet and inches / Meters and centimetres |
|  | 1. Height in feet: | ___ ft and ___inches |
|  | 1. Height in cm: | ___m and ___cm |
| **Baseline health** | 1. Do you have a family physician/primary care provider? | No /, Yes / I don't know |
|  | 1. Did you get a flu shot in the past year? | Yes / No |
|  | 1. Have you been diagnosed by a physician with any of the following chronic medical conditions? 2. Hypertension 3. Diabetes 4. Asthma 5. Chronic lung disease 6. Chronic heart disease 7. Chronic kidney disease 8. Liver disease 9. Cancer 10. Chronic blood disorder 11. Immune suppressed 12. Chronic neurological disorder | Yes / No |
|  | 1. Do you currently smoke tobacco? | Yes/ No |
|  | 1. How often do you smoke tobacco? | Less than daily / Daily |
|  | 1. Do you currently use e-cigarettes (vape)? | Yes/ No |
|  | 1. How often do you use e-cigarettes (vape)? | Less than daily / Daily |
| **COVID-19 exposure** | 1. Do you think you have had COVID-19? | Yes / No |
|  | 1. Why do you think you have had COVID-19 (select all that apply)? | Self-assessment online; symptom profile; nasal/throat test result; Healthcare provider; Contact with a COVID-19 case |
|  | 1. Were you hospitalized due to COVID-19? | Yes / No |
|  | 1. Has anyone in your household (not counting yourself) ever tested positive for COVID-19? | Yes / Not applicable, I live alone / No one has been tested / No, they tested negative / Not sure, waiting for the result |
|  | 1. Has anyone outside of your household, but with whom you've had close contact (within 2 meters and for 2 minutes or longer) ever tested positive for COVID-19? | No, not that I am aware of / Yes |
|  | 1. Who tested positive? (check all that apply) | A family member from outside my household / Friend / Co-worker / Student / Someone else, please list relationship |
|  | 1. Have you been tested for COVID-19? | Yes / No |
|  | 1. How many times have you been tested? | 1 / 2 / 3 / 4 / Five or more |
|  | 1. What was the date and result of each test?  \|  \| Date \| Result \| \| --- \| --- \| --- \| \| First test \|  \| Negative / Positive / I don't know \| \| Second test \|  \| Negative / Positive / I don't know \| \| Third test \|  \| Negative / Positive / I don't know \| \| Fourth test \|  \| Negative / Positive / I don't know \| |  |
|  | 1. Did you have any of the following symptoms when you got tested for COVID-19? 2. Cough 3. Fever 4. Shortness of breath 5. Sore muscles 6. Headache 7. Sore throat 8. Diarrhea 9. Decreased sense of smell 10. Other symptoms | Yes / No / I don’t know |
|  | 1. What was the date of your first symptom? | **____** |
| **COVID-19 related behaviour** | 1. Have you travelled outside your home province since January 2020? | Yes / No |
|  | 1. What province or country did you travel to (select all that apply)? | Alberta / British Columbia / Manitoba / New Brunswick / Newfoundland and Labrador /Northwest territories / Nova Scotia / Nunavut / Ontario / Prince Edward Island / Quebec / Saskatchewan / Yukon / United States of America / Mexico / France / Italy / China / India / Iran / United Kingdom / Other (Please Specify) |
|  | 1. How many times have you been in a gathering of 10 or more people over the past three months? | **___** |
|  | 1. How often have you done the following in the past three months? 2. Worn a mask in public places 3. Practiced physical distancing in public places 4. Avoided crowded places/gatherings 5. Avoided common greetings, like handshakes 6. Limited contact with people at higher risk (e.g., an elderly relative) 7. Self-isolated because you thought you were infected with COVID-19 8. Self-quarantined because you may have been exposed to COVID-19, but did not show symptoms | Never / Rarely / Occasionally / Often / Always |
|  | 1. In the past 3 months, did you attend any of the following activities in which there were five (5) or more people in attendance? 2. Meeting, conference (including at work) 3. Eaten at an indoor restaurant 4. Eaten at an outdoor restaurant 5. Visited a bar or nightclub 6. Indoor gathering of family or friends 7. Outdoor gathering of family or friends 8. Religious gathering, wedding, funeral, etc. 9. Gym, exercise class, team sport 10. Theatre, movie, opera, sporting event, casino 11. Other activity (Please specify) | Yes / No |
|  | 1. To the best of your knowledge, how often do your co-workers... 2. Physically distance from you 3. Wear a mask in your presence 4. Cover coughs 5. Wash hands, thoroughly and regularly 6. Stay home when they have symptoms, even if they are mild | Always / usually / Occasionally / Never |
|  | 1. To the best of your knowledge, how often do students in your school... 2. Physically distance from you 3. Wear a mask in your presence 4. Cover coughs 5. Wash hands, thoroughly and regularly 6. Stay home when they have symptoms, even if they are mild | Always / usually / Occasionally / Never |
| **COVID-19 Vaccine** | 1. Have you been vaccinated against COVID-19? | Yes / No |
|  | 1. How many doses of the COVID-19 vaccine have you received so far? | One dose / Two doses / More than two doses |
|  | 1. When did you receive your first dose of the COVID-19 vaccine? | **____** |
|  | 1. When did you receive your second dose of the COVID-19 vaccine? | **­­­­­­____** |
|  | 1. Which vaccine did you receive for your first dose? | Pfizer and BioNTech mRNA vaccine / Moderna mRNA vaccine / AstraZeneca Oxford vaccine / I don't know |
|  | 1. Which vaccine did you receive for your second dose? | Pfizer and BioNTech mRNA vaccine / Moderna mRNA vaccine / AstraZeneca Oxford vaccine / I don't know |
| **MENTAL HEALTH & PSYCOSOCIAL QUESTIONNAIRE** | |  |
| **Receptivity to vaccines** | 1. The following statements relate to **routine vaccines** (e.g., preschool and school aged vaccines such as measles and HPV; adult vaccines such as tetanus; older adult vaccines such as pneumonia). Please let us know if you agree or disagree with the following: 2. I am completely confident that routine vaccines are safe 3. Routine vaccination is unnecessary because vaccine-preventable diseases are not common anymore 4. When everyone else is vaccinated, I don’t have to get vaccinated too. 5. People should be vaccinated to prevent the spread of disease in the community 6. When I think about getting vaccinated, I weigh the benefits and risks to make the best decision possible. 7. Everyday stress (such as competing priorities or many demands on my time) prevents me from getting vaccinated. 8. Vaccines are effective | Strongly disagree; Disagree; Neither agree nor disagree; Agree; Strongly agree |
| **Receptivity to COVID-19 vaccine** | 1. The following statements relate to the COVID-19 vaccine. Please let us know if you agree or disagree with the following: 2. I am completely confident that the COVID-19 vaccine(s) that will be available in Canada will be safe 3. Vaccination against COVID-19 is unnecessary because the risk of getting the COVID-19 virus in Canada is small 4. If everyone else gets vaccinated with the COVID-19 vaccine, I won’t have to get vaccinated against COVID-19 too 5. People should be vaccinated against COVID-19 to prevent the spread of disease in the community 6. When I think about getting the COVID-19 vaccine, I will weigh benefits and risks to make the best decision possible 7. Everyday stress (such as competing priorities or many demands on my time) will prevent me from getting the COVID-19 vaccine | Strongly disagree; Disagree; Neither agree nor disagree; Agree; Strongly agree |
| **Intention to receive COVID-19 vaccine** | 1. If a vaccine becomes available and is recommended for me, I would get it | Yes / No / Unsure |
| **Urgency to receive COVID-19 vaccine** | 1. If I were to get vaccinated, I would get it as soon as it would be available to me | Yes, right away ;Yes, but I would wait a little bit; No, I don’t plan on getting it; Unsure |
| **Recommended by Experts** | 1. I would get the COVID-19 vaccine if public health experts recommend it | Yes / No / Maybe |
|  | 1. I would get the COVID-19 vaccine if my healthcare provider recommends it | Yes / No / Maybe |
|  | 1. I would get the COVID-19 vaccine if the government recommends it | Yes / No / Maybe |
| **Perceived benefits** | 1. Receiving the COVID-19 vaccine would: 2. Protect me from getting COVID-19 3. Protect my family from getting COVID-19 4. End the pandemic and make us return to normal life | Yes/ No/ Maybe |
| **Perceived susceptibility** | 1. I am at risk of getting Covid-19 | Strongly disagree; Disagree; Neither agree nor disagree; Agree; Strongly agree |
|  | 1. Someone in my family is at risk of getting Covid-19 |  |
|  | 1. I am at risk of severe complications from Covid-19 |  |
|  | 1. Someone in my family is at risk of getting really sick from Covid-19 |  |
| **Severity** | 1. Covid-19 is a serious disease | Strongly disagree; Disagree; Neither agree nor disagree; Agree; Strongly agree |
|  | 1. People can die if they get Covid-19 |  |
|  | 64) People who have mild symptoms for Covid-19, can still have long term health effects |  |
| **Barriers to getting the COVID-19 vaccine** | 1. The following may prevent me from getting the vaccine: 2. It may have serious short term side effects 3. It may have long term effects that we are unaware of 4. We do not know whether it will protect us for a long time | Strongly disagree; Disagree; Neither agree nor disagree; Agree; Strongly agree |
| **Denialism** | 1. Much of the information we receive about COVID-19 is wrong | Strongly disagree; Disagree; Neither agree nor disagree; Agree; Strongly agree |
|  | 1. I think health officials often hide the truth about COVID-19 |  |
|  | 1. Official government accounts of COVID-19 cannot be trusted |  |
| **General conspiracy** | 1. I believe the coronavirus was created in a laboratory according to plans unknown to the public | Strongly disagree; Disagree; Neither agree nor disagree; Agree; Strongly agree |
|  | 1. I believe there are groups interested in spreading panic about COVID-19 to achieve their own goals |  |
|  | 1. Many very important things happen in the world, which the public is never informed about | certainly not true (1) - certainly true (9) |
|  | 1. Politicians usually do not tell us the true motives in their decisions |  |
|  | 1. Events which superficially seem to lack a connection are often the result of secret activities |  |
|  | 1. There are secret organizations that greatly influence political decisions |  |
| **Mental Health** | 1. In general, how would you describe your mental health? | Excellent, Very good, Good, Fair; Poor |
| **Quality of Life** | 1. In general, would you say your quality of life is | Excellent, Very good, Good, Fair; Poor |
| **Anxiety**  GAD-7 Anxiety | 1. Over the **last two weeks**, how often have you been bothered by the following problems? 2. Feeling nervous, anxious, or on edge 3. Not being able to stop or control worrying 4. Worrying too much about different things 5. Trouble relaxing 6. Being so restless that it is hard to sit still 7. Becoming easily annoyed or irritable 8. Feeling afraid, as if something awful might happen | Not at all; Several days; More than half the days; Nearly every day |
| **Nonspecific distress** Kessler K6+ | 1. During the past 30 days, about how often did you feel: 2. Nervous 3. Hopeless 4. Restless or fidgety 5. So depressed that nothing could cheer you up 6. That everything was an effort 7. Worthless | All of the time; Most of the time; Some of the time; A little of the time; None of the time |
| **Optimism** | 1. How much do you agree or disagree with these questions? 2. In uncertain times, I usually expect the best. 3. If something can go wrong for me, it will. 4. I'm always optimistic about my future 5. I hardly ever expect things to go my way 6. I rarely count on good things happening to me. 7. Overall, I expect more good things to happen to me than bad. | I disagree a lot; I disagree a little; I neither agree nor disagree; I agree a little; I agree a lot |
| **Living situation** | 1. Currently, I live (Please choose all that apply): | On my own; With my partner/spouse; With child(ren) under 18 years of age; With adult children 18 years of age and older; With other adult relatives; With adult non-relatives |
| **Health behaviours** | 1. Since COVID-19, have your weekly habits changed for any of the following: 2. Exercise 3. Screen time outside of work (watching movies, spending time on the internet, playing video games, etc..) 4. Consumption of Fruits and Vegetables (not including juice or fried potatoes) 5. Eating sweet or salty treats 6. Drinking alcohol 7. Sleep quality | Increased, no change, decreased, Not applicable |
| **Dietary behaviours** | ***The next questions ask about how many servings of fruits and vegetables you ate yesterday. Use the examples below as a guide when you answer these questions.***   \| **1 serving of FRUIT could be:**   - 1 medium apple or orange - 1 medium banana - 1 large plum - ½ cup of berries - 20 cherries - 20 grapes - ¼ cup of dried fruit \| **1 serving of VEGETABLES could be:**   - ½ cup of broccoli - 1 large carrot - 1 cup of raw spinach - 1 cup of raw lettuce - ½ cup of sweet potatoes - ½ cup of green beans - 4 Brussel sprouts \| \| --- \| --- \| |  |
|  | 1. Yesterday, how many servings of fruit (including fresh, frozen, canned, and dried fruit) did you eat? DO NOT count 100% fruit juice. [None – 4 or more servings (in 0.5 serving increments)] | _______ |
|  | 1. Yesterday, how many servings of vegetables (including fresh, frozen or canned) did you eat? DO NOT count 100% vegetable juice or fried potatoes. [None – 4 or more servings (in 0.5 serving increments)] | _______ |
|  | 1. In the past week, how many alcoholic drinks did you consume 1 drink equals **a 12 ounce can or glass of beer or cooler, a 5 ounce glass of wine, or a drink containing 1 shot of liquor)** | 0, 1-2, 3-4, 5-6, 7-8 (1 a day), 9-10, 11-12, 13-14 (2 a day), 15-16, 17+ |
|  | 1. In the past week, how many servings of sweet or salty treats did you consume (1 serving = ½ cup of ice cream, 1 cup of chips, 2 small cookies, 1 brownie or piece of cake, a handful of candy, 1 can or cup of soda or sugary beverage)? | 0, 1-2, 3-4, 5-6, 7-8 (1 a day), 9-10, 11-12, 13-14 (2 a day), 15-16, 17+ |
| **PA behaviour**  **IPAQ Short** | **Please think about the activities you do at work, as part of your house and yard work, to get from place to place, and in your spare time for recreation, exercise or sport.** | |
|  | 1. Think about all the vigorous activities that you did in the last 7 days. Vigorous physical activities refer to activities that take hard physical effort and make you breathe much harder than normal. Think only about those physical activities that you did for at least 10 minutes at a time. During the last 7 days, on how many days did you do vigorous physical activities like heavy lifting, digging, aerobics, running or fast bicycling? | - 1. days |
|  | 86a) If > 0 days ask, How much time did you usually spend doing vigorous physical activities on one of those days? | _____ hours per day _____ minutes per day/Don’t know/Not sure |
|  | 1. Think about all the moderate activities that you did in the last 7 days. Moderate activities refer to activities that take moderate physical effort and make you breathe somewhat harder than normal. Think only about those physical activities that you did for at least 10 minutes at a time. During the last 7 days, on how many days did you do moderate physical activities like carrying light loads, or bicycling at a regular pace? Do not include walking. | - 1. days |
|  | 87a) If > 0 days ask, How much time did you usually spend doing moderate physical activities on one of those days? | _____ hours per day _____ minutes per day/Don’t know/Not sure |
|  | 1. Think about the time you spent walking in the last 7 days. This includes at work and at home, walking to travel from place to place, and any other walking that you have done solely for recreation, sport, exercise, or leisure. 5. During the last 7 days, on how many days did you walk for at least 10 minutes at a time? | - 1. days |
|  | 88a) If > 0 days ask, How much time did you usually spend walking on one of those days? | _____ hours per day _____ minutes per day/Don’t know/Not sure |
